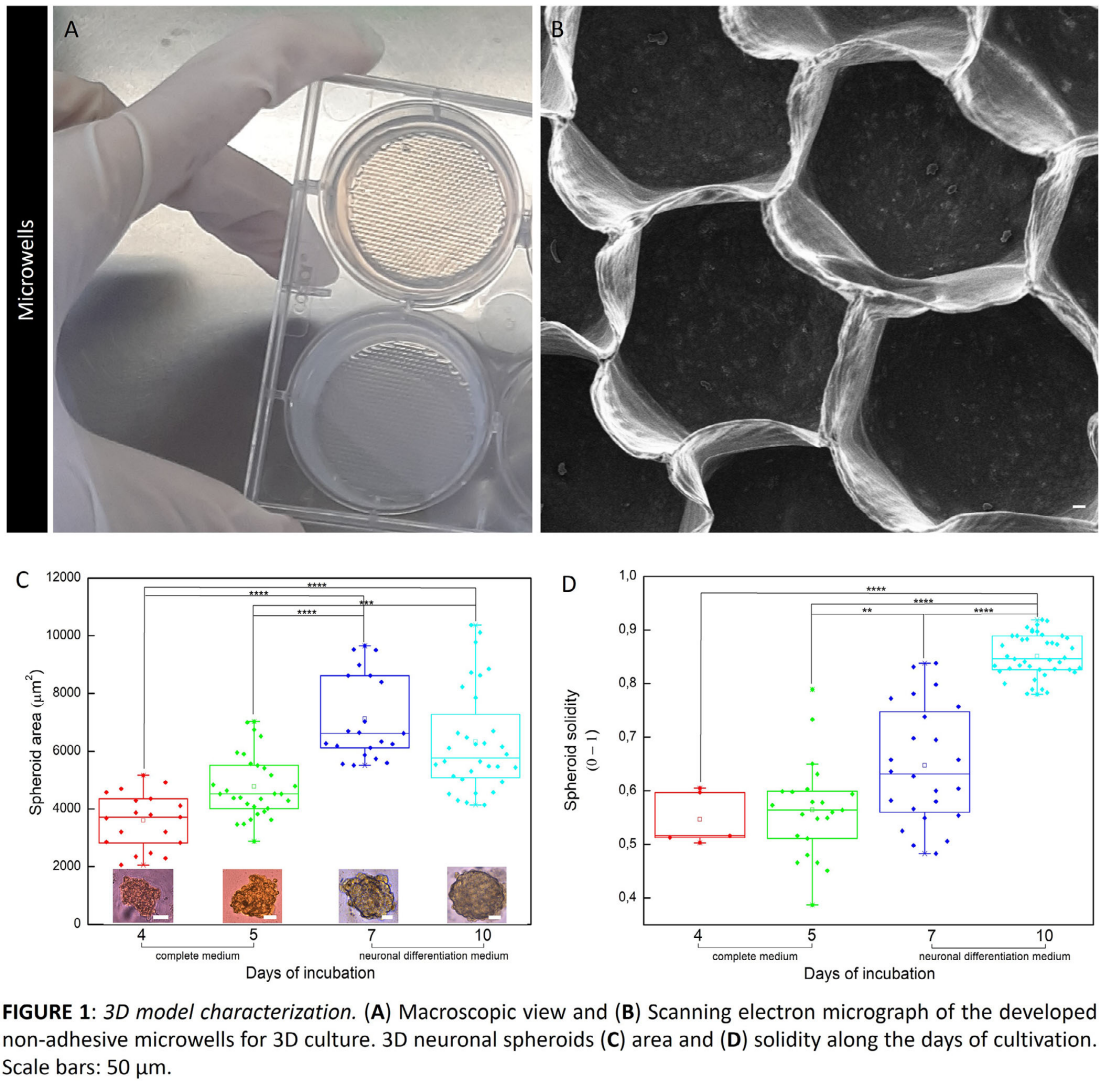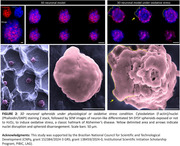# Neuronal spheroids under oxidative stress as a three‐dimensional model of Alzheimer's disease

**DOI:** 10.1002/alz70855_103750

**Published:** 2025-12-24

**Authors:** Geisa Rodrigues Salles, Luiza de Andrade Giraldi, Marimelia Aparecida Porcionatto, Cristina Pacheco‐Soares

**Affiliations:** ^1^ Universidade do Vale do Paraíba, São José dos Campos, São Paulo, Brazil; ^2^ INCT Model3D, São Paulo, Brazil; ^3^ Universidade Federal de São Paulo, São Paulo, Brazil

## Abstract

**Background:**

Modelling neurobiological events is crucial for understanding physiological and pathological conditions. Three‐dimensional (3D) spheroids are in the spotlight of modelling, sustainably contributing to reducing animals use in science and replicating specific neuronal mechanisms, such as neurite outgrowth, cellular interaction, and oxidative stress responses, elemental aspects to be evaluated in Alzheimer's disease (AD). This study aimed to produce a 3D human neuronal model of AD, by developing spheroids of neuron‐like cells under oxidative stress.

**Method:**

SH‐SY5Y (cells from human neuroblastoma) spheroids were self‐assembly‐arranged by cultivation on developed non‐adhesive microwells (morphologically characterized by scanning electron microscopy, SEM), under complete medium (for 5 days), followed by neuronal phenotype differentiation medium (for another 5 days). Spheroids were periodically imaged on a phase contrast microscope; their respective area and solidity were measured. On day 10 of cultivation, spheroids were exposed or not to oxygen peroxide (H_2_O_2,_ 200 µM, 1h), to mimic the classic neurotoxic event of oxidative stress in AD. Spheroids were fixed and stained for cytoskeleton (f‐actin)/nuclei (phalloidin/DAPI) and imaged by a confocal laser scanning microscope or SEM.

**Result:**

The produced microwells are homogeneous and robust and the spheroids are well‐structured on them (Figure 1A‐B). The area of the spheroids statistically increases for up to 7 days of cultivation, however, there is no statistic difference between day 7 and day 10, indicating that differentiation medium may have reduced cell proliferation, inducing cells to the neuronal phenotype (Figure 1C). Moreover, on day 10, spheroids are statistically more solid than in the previous days, demonstrating that structural integrity and compaction are enhanced along the days (Figure 1D). As shown in Figure 2, on day 10, the 3D models have neurite distribution along the whole composition, however, under oxidative stress, nuclei abnormal staining is observed surrounding the spheroids, which is also observed in SEM micrographs, representing an atypical organization and nuclear envelope disruption. There is also a clear morphological disarrangement of spheroids after H_2_O_2_‐exposure, which will be further studied regarding AD neurodegenerative involved pathways.

**Conclusion:**

The produced neuronal spheroids represent a promising 3D model to be used for therapeutic purposes and to contribute with elucidating complex cellular dynamics and pathways of AD.